# Value of conventional magnetic resonance imaging texture analysis in the differential diagnosis of benign and borderline/malignant phyllodes tumors of the breast

**DOI:** 10.1186/s40644-021-00398-3

**Published:** 2021-03-12

**Authors:** Xiaoguang Li, Nianping Jiang, Chunlai Zhang, Xiangguo Luo, Peng Zhong, Jingqin Fang

**Affiliations:** 1 Department of Radiology, Daping Hospital, Army Medical University, Chongqing, 400042 China; 2 Department of Pathology, Daping Hospital, Army Medical University, Chongqing, 400042 China

**Keywords:** Phyllodes tumors, Magnetic resonance imaging, Texture analysis, Differential diagnosis

## Abstract

**Background:**

The purpose of this study was to determine the potential value of magnetic resonance imaging (MRI) texture analysis (TA) in differentiating between benign and borderline/malignant phyllodes tumors of the breast.

**Methods:**

The preoperative MRI data of 25 patients with benign phyllodes tumors (BPTs) and 19 patients with borderline/malignant phyllodes tumors (BMPTs) were retrospectively analyzed. A gray-level histogram and gray-level cooccurrence matrix (GLCM) were used for TA with fat-suppressed T2-weighted imaging (FS-T2WI), diffusion-weighted imaging (DWI), apparent diffusion coefficient (ADC) images, and 2- and 7-min postcontrast T1W images on dynamic contrast-enhanced MRI (DCE-T1WI_2min_ and DCE-T1WI_7min_) between BPTs and BMPTs. Independent sample t-test and Mann-Whitney U test were performed for intergroup comparison. A regression model was established by using binary logistic regression analysis, and receiver operating characteristic (ROC) curve analysis was carried out to evaluate diagnostic efficiency.

**Results:**

For ADC images, the texture parameters angular second moment (ASM), correlation, contrast, entropy and the minimum gray values of ADC images (ADC_Minimum_) showed significant differences between the BPT group and BMPT group (all p<0.05). The parameter entropy of FS-T2WI and the maximum gray values and kurtosis of the tumor solid region of DCE-T1WI_7min_ also showed significant differences between these two groups. Except for ADC_Minimum_, angular second moment of FS-T2WI (FS-T2WI_ASM_), and the maximum gray values of DCE-T1WI_7min_ (DCE-T1WI_7min-Maximum_) of the tumor solid region, the AUC values of other positive texture parameters mentioned above were greater than 0.75. Binary logistic regression analysis demonstrated that the contrast of ADC images (ADC_Contrast_) and entropy of FS-T2WI (FS-T2WI_Entropy_) could be considered independent texture variables for the differential diagnosis of BPTs and BMPTs. Combined, the AUC of these parameters was 0.891 (95% CI: 0.793–0.988), with a sensitivity of 84.2% and a specificity of up to 89.0%.

**Conclusion:**

Texture analysis could be helpful in improving the diagnostic efficacy of conventional MR images in differentiating BPTs and BMPTs.

## Introduction

Phyllodes tumors (PTs) are rare breast fibroepithelial tumors and are classified as benign, borderline and malignant according to the characteristics of the stromal components [1]. Each of these three grades is considered to have a very different biological behavior. Surgery is a fundamental treatment for PTs, and different surgical approaches are commonly selected based on the histologic grade of the tumor [2–4]. In the clinic, BPTs are usually treated with local excision, while extensive excision, including mastectomy, is necessary to reduce recurrence for BMPTs [5, 6]. Therefore, an accurate preoperative diagnosis and qualitative grading are conducive to the selection of the surgical procedure. Biopsy is the basis of an accurate preoperative diagnosis for breast diseases. However, due to the complex composition and obvious heterogeneity of PTs, it is difficult to obtain representative tissues by percutaneous needle biopsy alone, which could lead to low accuracy in the pathological diagnosis [7].

Magnetic resonance imaging (MRI), with its high sensitivity and relatively high specificity, has become an important imaging method in the diagnosis of breast diseases. However, previous studies have found that the conventional MRI findings of BPTs and BMPTs overlap [8, 9], and it is very difficult for physicians to subjectively identify them without obvious morphological features. Current studies on the diagnostic grading ability of functional MRI parameters, such as ADC value, for PT grading are contradictory [10, 11], and DCE-MRI findings, such as enhancement pattern or TIC, are not of great value in predicting the histologic grade of PTs [11, 12]. In addition, MR spectroscopy, which can reflect tumor metabolites, has been unable to conclusively distinguish benign from borderline or malignant PTs to date [8]. Therefore, it is of importance to improve the diagnostic performance of MRI by changing the existing image analysis methods.

Texture analysis (TA) is a method used for the quantitative analysis of image grayscale distribution features and the relationship between pixels and spatial features. Compared with conventional imaging methods, TA can provide objective and additional quantitative image information on lesions independent of the subjective judgment and experience of clinicians or radiologists, adding potential clinical value [13]. Recently, computer-aided TA has been used for the diagnosis and treatment response and prognostic evaluations of cancer patients [14]. However, few studies have used conventional MRI TA to grade PTs. The purpose of this study was to determine the diagnostic performance of conventional MRI TA in differentiating between BPTs and BMPTs.

## Methods

### Patients

**Fig. 1:**
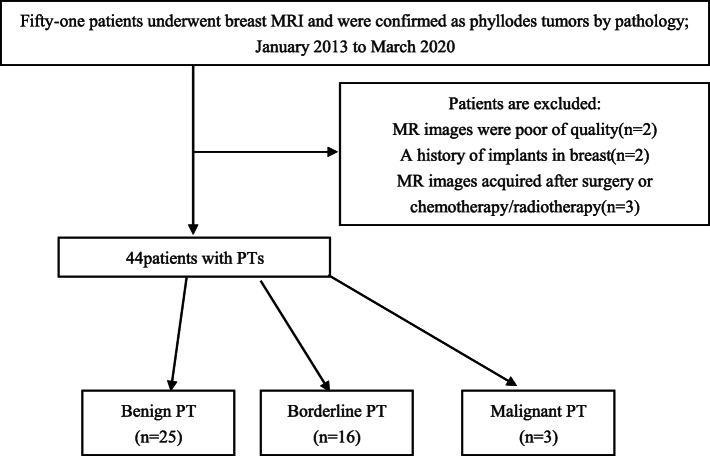
Flowchart for the case accrual process

We retrospectively reviewed the MRI data of fifty-one patients with surgically proven primary PTs admitted to our hospital between January 2013 and March 2020. 44 patients were enrolled in this study. The exclusion criteria included (1) MRI images with poor quality; (2) implants in one or both breasts; and (3) MRI images acquired after surgery, chemotherapy or radiotherapy. The patient ages ranged from 31 to 75 years (mean 48.55 ± 10.75 years). There were 25 cases of BPTs, 16 cases of borderline PTs, and 3 cases of malignant PTs based on the pathological results. The patient flowchart is illustrated in Fig. 1.

### Imaging protocol

All patients were examined with a 1.5 T MRI scanner (Siemens Magnetom Aera, Germany) in the standard prone position using an 8-channel dedicated breast coil. Axial T1WI (SE, TR = 8.6 ms, TE = 4.7 ms) and fat-suppressed T2WI (FSE, TR = 5600 ms, TE = 57 ms) were obtained. DWI was performed with a spin echo-echo planar imaging (SE-EPI) sequence with two b values (0 and 1000 s/mm^2^) in 3 orthogonal directions. The imaging parameters were as follows: TR = 3300 ms, TE = 94 ms, flip angle = 90°, layer thickness = 5 mm, matrix = 128 × 128, and FOV = 320 mm × 320 mm. Following DWI, DCE-MRI was performed with a 3D fat-suppressed T1 fast-field echo sequence (TR = 4.62 ms, TE = 1.75 ms, layer thickness = 1.5 mm, interlayer spacing = 0, FOV = 360 mm × 360 mm, and matrix = 384 × 320) before and five times after the injection of 0.1 mmol/kg gadopentetate dimeglumine (Omniscan, GE Healthcare, Ireland). Subsequently, 7-phase DCE-T1W images were acquired.

### Imaging analysis

**Fig. 2:**
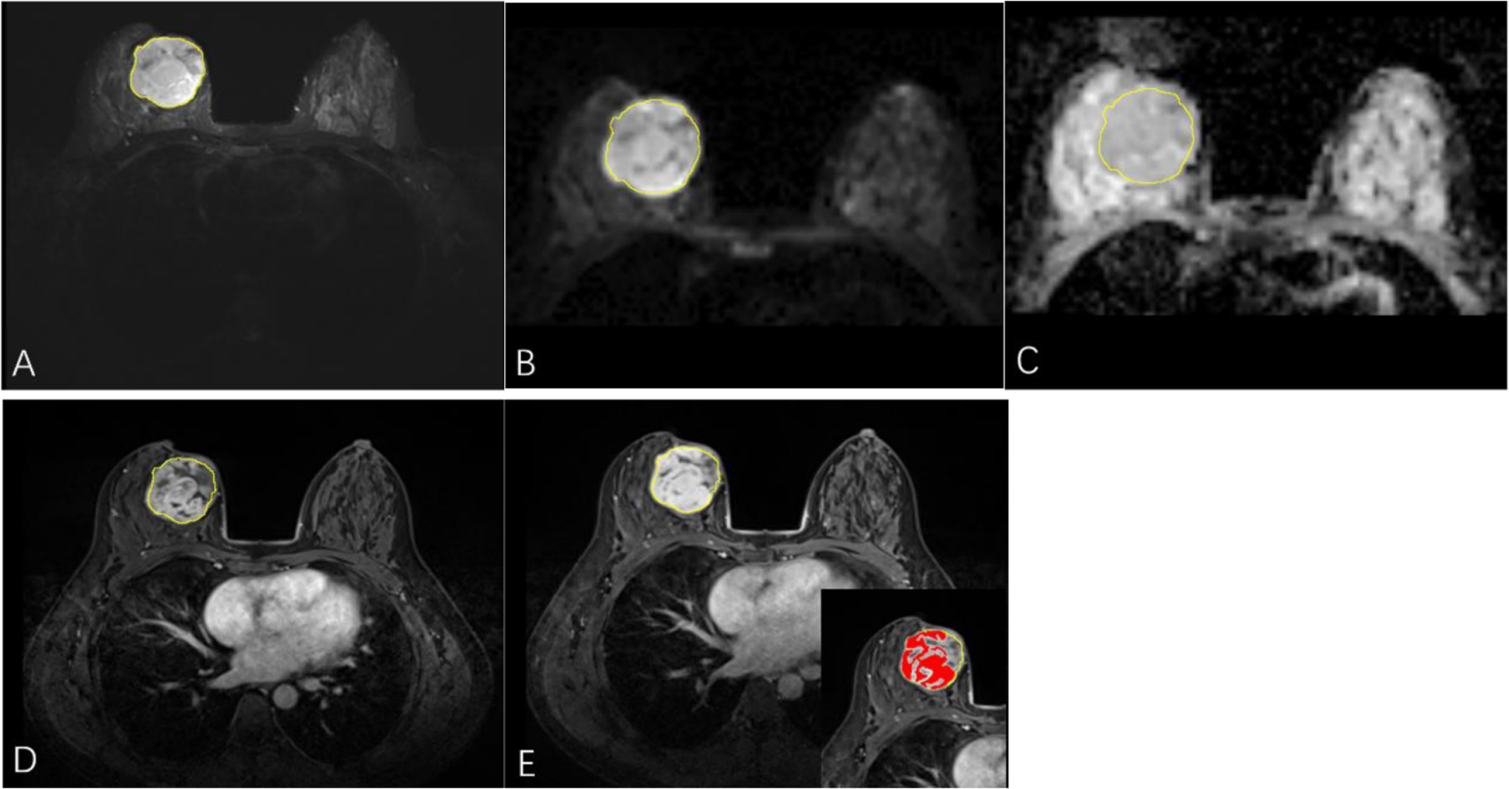
Schematic diagram of the ROI. ImageJ software was used to select the layer with the maximum tumor are from the FS-T2WI (**a**) and manually delineate the tumor boundary as much as possible, which was automatically copied to the DWI (**b**), ADC image (**c**), DCE-T1WI_2min_ (**d**), and DCE-T1WI_7min_ (**e**) of the same tumor layer. Note that the red part in the lower right corner of the DCE-T1WI_7min_ (**e**) represents the solid tumor region

All FS-T2WI, DWI, ADC, DCE-T1WI_2min_ and DCE-T1WI_7min_ tumor images were exported in DICOM format from the PACS system and then imported to RadiAnt software (https://www.radiantviewer.com/) to render them in BMP format with identical window widths and window levels. ImageJ software (https://rsb.info.nih.gov/ij/) was used for image TA (Fig. 1), and all the data were analyzed separately by two radiologists (CL.Z. and XG.L., with 10 and 15 years of experience in breast imaging, respectively). The regions of interest (ROIs) were extracted as follows: ROIs were placed on the image containing the maximal tumor area for all MR images and included necrotic, cystic, and hemorrhagic areas. The tumor solid region was delineated on DCE-T1WI_7min_ (Fig. 2). Finally, the gray-level histogram and gray-level cooccurrence matrix (GLCM) parameters of all the ROIs were automatically measured by the software [15]. Definitions and formulas for the histogram and GLCM parameters are shown in Table 1. GLCM is a spatial domain statistical technique that calculates second- and higher-order statistics for the number of paired (*i*, *j*) occurrences for which a gray level *i* is spaced away from a gray level *j* by a distance (d) and along a direction (θ) [16]. In this study, the relationship between the pixels of the GLCMs was set with d = 1 and θ =0° [17, 18]. The final histogram and GLCM parameter values of each lesion were the mean of the measured results from the two radiologists.

**Table 1:** Representative gray-level histogram and gray-level co-occurrence matrix texture features

Texture parameters	Qualitative description	Mathematical description
Histogram parameters		
Mean	Mean gray-level value	Mean= $\sum k\frac{k^{\ast }g(k)}{\sum k{g}^k}$
Minimum	Minimum gray-level value	Min= *Min*(*k*)
Maximum	Maximum gray-level value	Max= *Max*(*k*)
Skewness	Measure of histogram symmetry	Skew= σ^−4^∑_*k*_(*k* − μ)^4^ ∗ g(k) − 3
Kurtosis	Measure of histogram flatness	Kurt= σ^−3^∑_*k*_(*k* − μ)^4^ ∗ g(k)
GLCM parameters		
Angular Second Moment (ASM)	Certainty of gray-level co-occurrence	ASM = ${\sum}_{i,j}f{\left(i,j\right)}^2$,
Contrast	Intensity contrast between pixel and its neighbor	CON = ${\sum}_{i,j}\left|i\right.-{\left.j\right|}^2f\left(i,j\right)$
Correlation	Linear gray-level dependence	COR = ${\sum}_{i,j}\frac{{\left(i-{\mu}_i\right)}^{\ast }{\left(j-{\mu}_j\right)}^{\ast }f\left(i,j\right)}{\left(1+{\left(i-j\right)}^2\right)}$
Inverse difference moment (IDM)	Local homogeneity in gray-level co-occurrence	IDM = ${\sum}_{i,j}\frac{f\left(i,j\right)}{\left(1+{\left(i-j\right)}^2\right)}$
Entropy	Uncertainty of gray-level co-occurrence	ENT = $-{\sum}_{i,j}f{\left(i,j\right)}^{\ast}\log \left(f\left(i,j\right)\right)$

### Statistical analysis

Statistical analyses were performed using IBM SPSS version 21.0 (IBM Corporation, New York). *Kolmogorov-Smirnov* and *Levene* tests were used to determine the normality and homogeneity of variance, respectively, of all measurement data. The independent sample *t*-test and the *Mann-Whitney U* test were used for data with normal and nonnormal distributions, respectively. *Bonferroni’s* correction was used to adjust *p* values for multiple parameter comparisons [19]. For texture parameters with significant differences, the group was taken as the dependent variable, logistic regression analysis was performed for multiparameter joint analysis, and the predicted value of the computational model was used to draw the receiver operator characteristic (ROC) curve. The efficacy (sensitivity, specificity, 95% confidence interval and *p* value) of each individual texture parameter and of the combined parameters in the identification of the two groups was determined with the maximum parameter value of the Youden index [(sensitivity + specificity)-1] as the threshold. *p* < 0.05 indicated a statistically significant difference.

## Results

### Comparisons of texture parameters of different images between the BPT and BMPT groups

As illustrated in Tables 2 and 3, for FS-T2WI, the GLCM texture parameters ASM and entropy were significantly different between the two groups (both *p*<0.05). However, no histogram parameters showed significant intergroup differences. For ADC images, the GLCM parameters ASM, correlation, contrast, entropy and histogram parameter ADC_Minimum_ showed significant differences (all p<0.05). For DWI and DCE-T1WI_2min_, none of the histogram or GLCM parameters showed significant differences (all p>0.05). For DCE-T1WI_7min_, none of the histogram or GLCM parameters of tumor overall region showed significant differences. The maximum gray values and kurtosis of the tumor solid region showed significant differences (all p<0.05); however, no GLCM parameters showed significant differences for this region of the tumor (all p>0.05).

**Table 2:** Comparisons of histogram parameters of ADC images, FS-T2WI, DWI, DCE-T1WI_2min_ and DCE-T1WI_7min_ between the two groups, respectively

Parameters	BPTs(*n* = 25)	BMPTs(*n* = 19)	P value	Adjusted *P* value
ADC_Mean_	174.790 ± 26.849	164.122 ± 31.669	0.234	0.229
ADC_Minimum_	138.154 ± 37.446	108.842 ± 38.312	0.022	0.018
ADC_Maximum_	216.240 ± 33.286	218.263 ± 29.790	0.836	0.833
ADC_Skewness_	0.097 ± 0.804	0.147 ± 0.858	0.844	0.842
ADC_kurtosis_	0.797 ± 1.553	0.988 ± 1.598	0.693	0.688
^a^ FS-T2WI_Mean_	131.680 (95.702,160.844)	147.528 (124.366,154.154)	0.387	0.568
FS-T2WI_Minimum_	50.600 ± 35.344	54.263 ± 32.527	0.726	0.722
^a^ FS-T2WI_Maximum_	234.000 (175.500,255.000)	244.000 (222.000, 255.000)	0.259	0.264
FS-T2WI_Skewness_	0.054 ± 0.571	0.228 ± 0.516	0.304	0.298
^a^ FS-T2WI_Kurtosis_	0.372 (0.097,1.701)	0.110(−0.318,0.918)	0.110	0.121
DWI_Mean_	166.476 ± 30.833	160.400 ± 36.953	0.556	0.549
DWI_Minimum_	93.640 ± 34.946	100.053 ± 33.766	0.544	0.538
^a^ DWI_Maximum_	245.000 (229.500,247.500)	245.000 (229.000,252.000)	0.601	0.893
^a^ DWI_Skewness_	−0.254(−0.441,0.385)	0.192(− 0.151,.443)	0.132	0.405
^a^ DWI_Kurtosis_	−0.152(− 0.511,0.503)	−0.102(− 0.523,0.463)	0.972	0.704
(DCE-T1WI_2min_)_Mean_	132.006 ± 35.1049	125.593 ± 36.500	0.558	0.552
^a^ (DCE-T1WI_2min_)_Minimum_	58.000 (45.000,91.500)	36.000 (25.000,50.000)	0.048	0.128
(DCE-T1WI_2min_)_Maximum_	200.320 ± 32.983	202.526 ± 39.141	0.840	0.838
(DCE-T1WI_2min_)_Skewness_	0.0409 ± 0.56225	−0.0359 ± 0.79452	0.709	0.704
^a^ (DCE-T1WI_2min_)_Kurtosis_	−0.041(− 0.380,0.813)	−0.018(− 0.706,0.789)	0.731	0.493
(DCE-T1WI_7min_)_Mean_	179.321 ± 35.092	166.457 ± 41.996	0.275	0.270
(DCE-T1WI_7min_)_Minimum_	91.080 ± 56.3116	76.632 ± 52.2007	0.389	0.383
^a^ (DCE-T1WI_7min_)_Maximum_	243.000 (232.000,250.500)	232.000 (214.000,241.000)	0.037	0.145
(DCE-T1WI_7min_)_Skewness_	−0.647 ± 0.708	−0.793 ± 0.811	0.527	0.521
^a^ (DCE-T1WI_7min_)_Kurtosis_	0.691(−0.229,1.932)	1.210 (0.285,2.972)	0.420	0.414
^a^ (DCE-T1WI_7min_)_Mean_ of tumor solid region	200.935 (173.433,211.289)	184.417 (150.868,206.929)	0.260	0.224
(DCE-T1WI_7min_)_Minimum_ of tumor solid region	142.080 ± 45.118	123.000 ± 41.919	0.160	0.157
^a^ (DCE-T1WI_7min_)_Maximum_ of tumor solid region	240.000 (231.500,247.000)	209.000 (231.000,238.000)	0.024	0.028
(DCE-T1WI_7min_)_Skewness_ of tumor solid region	−0.023 ± 0.6125	−0.282 ± 0.919	0.268	0.263
^a^ (DCE-T1WI_7min_)_Kurtosis_ of tumor solid region	0.161(−0.189,0.620)	1.178 (0.507,2.677)	0.004	0.009

Note: Plus-minus values are means ± SD. ^a^ The data were expressed as median (quantile range, QR), and intergroup comparison was analyzed with Mann-Whitney U text. ADC: apparent diffusion coefficient; *FS-T2WI* fat-suppressed T2-weighted imaging; *DWI* diffusion weighted imaging; *DCE-T1WI*_*2min*_ *and DCE-T1WI*_*7min*_ 2- and 7-min postcontrast T1W images on DCE-MRI; *ASM* angular second moment; *IDM* inverse difference moment

**Table 3:** Comparisons of GLCM parameters of ADC images, FS-T2WI, DWI, DCE-T1WI_2min_ and DCE-T1WI_7min_ between the two groups, respectively

Parameter	BPTs(n = 25)	BMPTs(n = 19)	P value	Adjusted P value
ADC_ASM_ (10^− 4^)	32.402 ± 20.370	17.242 ± 10.652	0.003	0.007
ADC_Contrast_	39.516 ± 15.473	71.30 ± 31.43	0.000	0.000
^a^ ADC_Correlation_	23.866 (13.465,40.467)	9.577 (5.320, 12.695)	0.002	0.004
ADC_IDM_ (10^− 4^)	0.273 ± 0.070	0.236 ± 0.045	0.056	0.057
ADC_Entropy_	6.356 ± 0.750	6.990 ± 0.542	0.003	0.005
^a^ FS-T2WI_ASM_ (10^−4^)	10.218 (7.374, 13.788)	6.054 (4.924, 8.588)	0.017	0.023
FS-T2WI_Contrast_	284.043 ± 163.702	262.016 ± 89.184	0.600	0.594
^a^ FS-T2WI_Correlation_	6.390 (3.824, 10.029)	4.255 (3.864, 5.710)	0.166	0.337
^a^ FS-T2WI_IDM_ (10^− 4^)	0.128 (0.096, 0.157)	0.126 (0.115, 0.153)	0.325	0.195
^a^ FS-T2WI_Entropy_	7.071 (6.760, 7.602)	7.745 (7.364, 7.990)	0.004	0.011
^a^ DWI_ASM_ (10^−4^)	10.573 (7.104, 17.995)	9.214 (7.660, 14.154)	0.822	0.913
DWI_Contrast_	127.468 ± 70.053	108.686 ± 78.431	0.407	0.401
^a^ DWI_Correlation_	4.785 (3.970, 6.325)	5.340 (4.159, 8.704)	0.374	0.781
DWI_IDM_ (10^−4^)	0.179 ± 0.080	0.193 ± 0.083	0.560	0.553
DWI_Entropy_	7.070 ± 0.627	7.176 ± 0.573	0.566	0.560
^a^ (DCE-T1WI_2min_)_ASM_ (10^−4^)	6.671 (4.723,8.487)	4.454 (3.295,9.788)	0.387	0.734
^a^ (DCE-T1WI_2min_)_Correlation_	147.562 (109.739,278.188)	142.800 (112.616,358.782)	0.785	0.477
^a^ (DCE-T1WI_2min_)_Contrast_	5.624 (3.773,10.021)	5.839 (4.304,9.361)	0.610	0.666
^a^ (DCE-T1WI_2min_)_IDM_ (10^−4^)	0.1319 (0.1108,0.1689)	0.137 (0.096,0.185)	0.731	0.816
(DCE-T1WI_2min_)_Entropy_	7.529 ± 0.678	7.741 ± 0.635	0.296	0.291
^a^ (DCE-T1WI_7min_)_ASM_ (10^−4^)	7.9483 (5.086,14.714)	9.201 (5.506,12.687)	0.661	0.730
^a^ (DCE-T1WI_7min_)_Correlation_	188.777 (151.258,350.693)	205.771 (43.797,296.260)	0.991	0.711
^a^ (DCE-T1WI_7min_)_Contrast_	3.668 (2.910,5.350)	4.623 (3.475,5.598)	0.222	0.191
(DCE-T1WI_7min_)_IDM_ (10^−4^)	0.169 ± .0708	0.171 ± 0.059	0.907	0.906
(DCE-T1WI_7min_)_Entropy_	7.360 ± 0.772	7.562 ± 0.610	0.354	0.348
^a^ (DCE-T1WI_7min_) _ASM_ of tumor solid region (10^−4^)	9.306 (5.4594,15.915)	10.669 (5.257,13.941)	0.400	0.561
^a^ (DCE-T1WI_7min_)_Correlation_ of tumor solid region	189.607 (129.702,350.693)	209.754 (141.136,296.260)	0.934	0.735
^a^ (DCE-T1WI_7min_)_Contrast_ of tumor solid region	3.772 (3.082,5.563)	4.712 (3.331,9.056)	0.314	0.222
(DCE-T1WI_7min_)_IDM_ of tumor solid region (10^−4^)	0.175 ± 0.074	0.169 ± 0.057	0.770	0.766
(DCE-T1WI_7min_)_Entropy_ of tumor solid region	7.230 ± 0.760	7.495 ± 0.654	0.232	0.227

Note: Plus-minus values are means ± SD. ^a^ The data were expressed as median (quantile range, QR), and intergroup comparison was analyzed with Mann-Whitney U text. *ADC* apparent diffusion coefficient; *FS-T2WI* fat-suppressed T2-weighted imaging; *DWI* diffusion weighted imaging; *DCE-T1WI*_*2min*_ *and DCE-T1WI*_*7min*_ 2- and 7-min postcontrast T1W images on DCE-MRI; *ASM* angular second moment; *IDM* inverse difference moment

### Diagnostic efficacy of MRI texture analysis in differentiating between BPTs and BMPTs

**Fig. 3:**
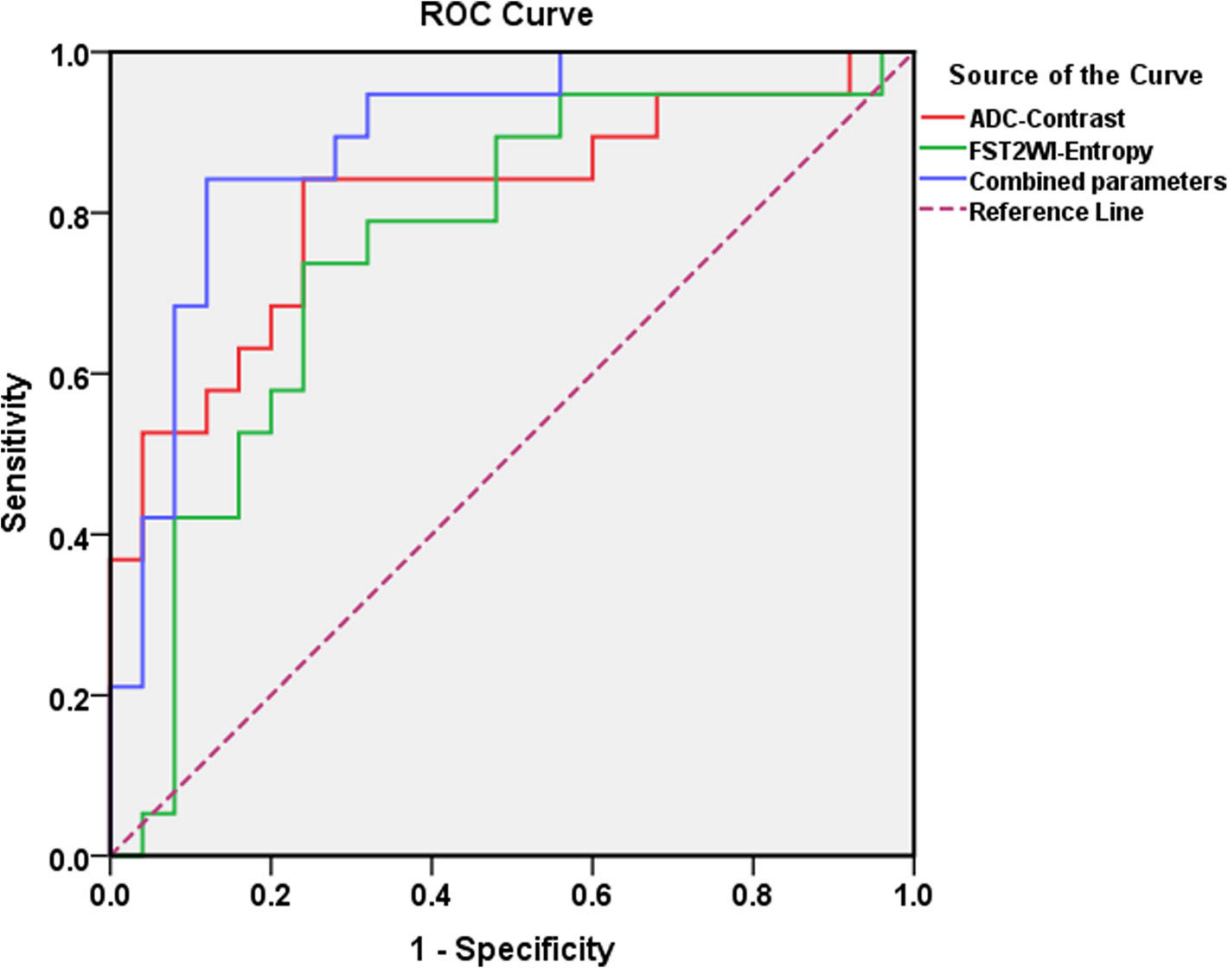
ROC curves of independent variables and the combination of texture parameters from the logistic regression model for differentiating BPTs from BMPTs

The parameters with significant differences between the two groups were further analyzed by ROC curve analysis. Those parameters with an AUC > 0.75 were ADC_ASM_, ADC_Contrast_, ADC_Correlation_, ADC_Entropy_, FS-T2WI_Entropy_, and kurtosis of DCE-T1WI_7min_ (DCE-T1WI_7min-Kurtosis_) of the tumor solid region. Among them, ADC_Contrast_ had the highest differential diagnostic efficiency, with an AUC of 0.815, a sensitivity of 84.2% and a specificity of 76.0%. Binary logistic regression analysis revealed that both ADC_Contrast_ and FS-T2WI_Entropy_ showed significant differences between the two groups (*p* < 0.05) and were thus regarded as independent variables. Then, the following regression eq. (RE) was obtained: *P = -13.616 + 0.067ADC*_*Contrast*_ *+ 1.341FS-T2WI*_*Entropy*_. The ROC curve of the combined texture parameters from the logistic regression model was plotted, and its identification efficiency was shown to be better than that of each individual texture parameter. The AUC was 0.891 (95% CI: 0.793–0.988), with a sensitivity of 84.2% and a specificity of up to 89.0% (Table 4, Fig. 3).

**Table 4:** Receiver operating characteristic curve analysis for the positive texture variables between the BPTs and BMPTs

Parameter	AUC	95%CI	Sensitivity	Specificity
ADC_Minimum_	0.705	0.504–0.829	70.4%	65.0%
ADC_ASM_	0.756	0.609–0.903	73.7%	72.0%
ADC_Contrast_	0.815	0.680–0.949	84.2%	76.0%
ADC_Correlation_	0.781	0.642–0.920	84.2%	72.0%
ADC_Entropy_	0.785	0.647–0.923	78.9%	76.0%
FS-T2WI_ASM_	0.712	0.546–0.877	73.7%	76.0%
FS-T2WI_Entroy_	0.756	0.607–0.905	73.7%	78.0%
DCE-T1WI_7min-Maximum_ of solid tumor region	0.701	0.544–0.858	78.9%	60.0%
DCE-T1WI_7min-Kurtosis_ of solid tumor region	0.758	0.600–0.916	78.9%	76.0%
Combined parameters	0.891	0.793–0.988	84.2%	89.0%

Note: *ADC* apparent diffusion coefficient; *FS-T2WI* fat-suppressed T2-weighted imaging; *ASM* angular second moment; *IDM* inverse difference moment. *DCE-T1WI*_*7min*_ 7-min postcontrast T1W images on DCE-MRI

## Discussion

TA is a radiomics technique that can help reveal the potential heterogeneity within tumor lesions and provide quantitative and objective information on conventional MR images in the clinic [20]. First-order TA is performed through the gray-level histogram, which mainly describes the distribution of individual gray intensity values. Generally, the ROI is decomposed into single values representing gray-signal intensity: the mean value, maximum value, minimum value, skewness, and kurtosis. A higher gray value indicates a brighter ROI area. The ADC image histogram is the most popular method for analyzing MRI tumor histograms, and a series of parameters obtained from the ADC image histogram for retrospective analysis have good repeatability [21]. In our study, both the ADC_Mean_ and ADC_Maximum_ gray values of BPTs were larger than those of BMPTs; however, there was no significant difference between these two groups, similar to the conclusion made in the study by Guo et al. [12]. In his study, there was no significant difference in the ADC values between the BPT and BMPT groups for the mean ROI-w (the whole-tumor ROI) values or for the 10th, 25th, 50th and 75th percentile values from the ROI-w histogram.

Previous studies have found that the minimum ADC value has the best accuracy in differentiating between malignant and benign breast masses [22, 23]. In this study, we found that the ADC_Minimum_ gray value of BPTs was significantly higher than that of BMPTs, which indicates that the ADC_Minimum_ gray value can better display areas with higher cellular density than the ADC_Mean_ gray value. The mean ADC value based on conventional hot spot ROIs or the histogram ROI only represents the average level of the data, which might be limited to PTs with considerable heterogeneity. However, it should be emphasized that the ADC_Minimum_ gray value may be more susceptible to outliers from noise, artifacts, adjacent structures and the partial volume effect; therefore, great care should be taken when delineating ROIs [24]. Kurtosis and skewness are statistics reflecting the distribution of the image gray values. The steeper the kurtosis, the steeper the distribution is compared with the normal distribution; the greater the absolute value of skewness, the greater the skewness of the distribution is [25, 26]. In our study, neither of these two parameters was able to distinguish BPTs from BMPTs, indicating that they are of little importance in distinguishing the two groups based on the morphological changes in ADC gray histograms.

The GLCM is one of the important methods used in second-order TA. The GLCM can describe the spatial relationship between voxels by analyzing the gray distribution of pixels and the surrounding spatial domain [27]. The texture parameter ASM reflects homogeneity, the value of which is quite high when the image has perfect homogeneity or when the pixel intensity is very similar. Correlation reflects the linear dependency of the gray levels of neighboring pixels, and high values can be obtained for regions of similar gray levels [28]. In this study, the GLCM derived from ADC images showed that the ASM and correlation values of BPTs were significantly higher than those of BMPTs. This indicates that BPTs have a more uniform gray distribution and stronger texture regularity than BMPTs on ADC images. Contrast reflects the amount of gray-level variation in an image, where a high contrast value indicates the presence of noise or a wrinkled texture in an image. The increased contrast values of BMPTs suggest more noise or wrinkled textures in malignant PT lesions, which may be associated with the local heterogeneous intensity. Entropy represents the amount of information needed for image compression. A higher entropy value represents a greater loss of image information and a more complex image texture [29]. In this study, BMPTs had a higher entropy value than BPTs, suggesting that BMPTs lose more image information and thereby have increased complexity and heterogeneity.

FS-T2WI is one of the more important sequences for MRI TA, which may be related to the relatively long time of echo (TE) of the sequence, increasing the interorganizational contrast and making the image contain more texture features of diagnostic significance [30]. In this study, we did not find any FS-T2WI gray histogram parameter that could distinguish BPTs from BMPTs. However, we did find significant differences in the GLCM parameters ASM and entropy between the two groups. The entropy of BPTs was significantly lower than that of BMPTs, indicating that the FS-T2WI texture of BMPTs is more complex and heterogeneous than that of BPTs. This may be related to the fact that BMPTs are more prone to allogenic metaplasia, which further complicates their internal composition. To some extent, these heterogeneous structures can also explain why the ASM of BMPTs was significantly lower than that of BPTs. It is worth mentioning that in empirical imaging analysis, we tend to consider that the degree of diffusion limitation of malignant PTs on DWI is more obvious and that the signal is higher than that of benign PTs. A previous study showed that the accuracy of DWI in characterizing lesions by using b values = 0 s/mm^2^ and 1000 s/mm^2^ was the best when breast lesions were identified on 1.5-T MRI [31]. Therefore we attempted to verify whether high b value (b = 1000 s/mm^2^) DWI TA could be of importance in differentiating between BPTs and BMPTs. However, the results were disappointing, showing that none of the histogram and GLCM parameters could differentiate between BPTs and BMPTs. We conjecture that this might be related to the nature of high b value DWI, in which the signal-to-noise ratio (SNR) can be reduced, along with image information.

Of the 7 phases of DCE-MRI scanning performed, we selected DCE-T1WI_2min_ and DCE-T1WI_7min_ for study, mainly because the contrast agent had just entered the tumor at 2 min of DCE-T1WI, and the texture comparisons were substantial; furthermore, at 7 min of DCE-T1WI, all components of the tumor could demonstrate significant contrast with the surrounding glandular tissues [32]. The results showed that the histograms of the parameters mean, minimum and maximum gray value of DCE-T1WI_2min_ and DCE-T1WI_7min_ (both solid and overall region) were higher in the BPT group than in the BMPT group, and only the maximum gray value of DCE-T1WI_7min_ for tumor solid region showed significant differences between the two groups after Bonferroni’s correction. This indicates a higher enhancement degree for the tumor solid region in BPTs than in BMPTs. Additionally, the kurtosis of the tumor solid region in the BPT group was significantly lower than that in the BMPT group, which suggests a more uniform signal from the tumor solid region in BPTs on DCE-T1WI_7min_. Previous studies [33] have shown that the GLCM based on DCE-MRI can better reflect tumor heterogeneity, and texture differences may reflect the potential pathological subtypes of breast cancer. However, we found no significant difference in GLCM parameters between the BPT and BMPT groups, either in the tumor overall region on DCE-T1WI_2min_ and DCE-T1WI_7min_ or in the tumor solid region on DCE-T1WI_7min_.

In this study, although multiple texture parameters were statistically significant in differentiating between BPTs and BMPTs, by drawing the ROC curves, we found that the GLCM parameters derived from ADC images had better diagnostic performance, in which the AUC of contrast reached more than 0.8, with the highest sensitivity (84.2%) and specificity (76.0%). Furthermore, binary logistic regression analysis showed that the texture parameters ADC_Contrast_ and FS-T2WI_Entropy_ were independent variables in differentiating BPTs from BMPTs. ROC curve analysis showed that the combination of these two texture parameters had excellent diagnostic efficiency, with an AUC of 0.891, an optimal sensitivity of 0.842 and a specificity of 0.890, all of which were better than the diagnostic efficiency of individual sequences and single parameters.

There are several limitations to our study that deserve discussion. First, it is inevitable that the size of the sample would be small, especially for malignant PTs. Second, this study was a single-center retrospective study with no external data validation, and in particular, the differences in MRI scanning protocols between different studies may lead to deviations in the image data. Third, only DWI based on b = 1000/mm^2^ were used for texture analysis, and other DWI with different b values should be examined in the future. Fourth, our study was performed using 1.5 T MRI systems for acquiring DWI, and the possibility of differing results using a higher magnetic field (3 T) cannot be excluded. Fifth, we analyzed only the two-dimensional features of the maximum surface of the tumor but did not obtain the three-dimensional features of the whole tumor. Sixth, only first-order histograms and second-order GLCM parameters were used for the differentiational diagnosis of PTs; whether higher-order texture parameters, such as the run-length matrix (RLM) and absolute gradient matrix (ARM), are helpful in the identification of BPTs and BMPTs is worth further discussion.

## Conclusion

This study conducted a texture analysis based on FS-T2WI, DWI (b = 1000/mm^2^), ADC images, DCE-T1WI_2min_ and DCE-T1WI_7min_ to explore their diagnostic value in the preoperative classification of PTs. The results showed that the texture parameters that could aid in the differentiation between BPTs and BMPTs were mainly derived from the GLCM analysis of ADC images, among which ADC_Contrast_ had the highest differential efficacy. In addition, we found that combined multiparameter TA from multiple images could greatly improve the efficiency of the identification of BPTs and BMPTs. Thus, MRI texture analysis may be used as an image-processing tool that is worthy of further evaluation in the differential diagnosis of BPTs and BMPTs.

## Data Availability

The datasets supporting the conclusions of this article are included within the article.

## Abbreviations

MRI: Magnetic resonance imaging
DCE-MRI: Dynamic contrast-enhanced MRI
FS-T2WI: T2-weighted imaging with fat suppression
DWI: Diffusion-weighted imaging
ADC: Apparent diffusion coefficient
TA: Texture analysis
BPTs: Benign phyllodes tumors
BMPTs: Borderline/malignant phyllodes tumors
ROC: Receiver operating characteristic
ROI: Region of interest
AUC: The area under the curve
GLCM: Gray-level cooccurrence matrix
ASM: Angular second moment
IDM: Inverse difference moment
